# The influence of age and gender on emu (*Dromaius novaehollandiae*) fat

**DOI:** 10.1038/s41598-020-68103-1

**Published:** 2020-07-06

**Authors:** Mateusz Bucław, Danuta Majewska, Danuta Szczerbińska, Marek Ligocki

**Affiliations:** Department of Monogastric Animal Sciences, Laboratory of Poultry Science, West Pomeranian University of Technology Szczecin, Janickiego Str. 29, 71-270 Szczecin, Poland

**Keywords:** Fats, Fatty acids

## Abstract

Studies were carried out to determine the influence of age and sex on two types of fat (back fat and abdominal fat) in the emu, as these are factors that influence the composition of animal tissues. The material involved 26 emus at the age 1 (6 males), 3 (6 males) and 15 years (8 females and 6 males), kept on the same farm and fed the feed of the same nutritional value. The basic chemical composition, cholesterol and mineral content, as well as fatty acid profile of back and abdominal fat of emu were determined. Abdominal fat was characterized by higher content of fat and ash, as well as Mn and Ba. Back fat, on the other hand, showed a higher level of protein, cholesterol, C16:1 and the elements K, P, Si, Na, Ca, Mg, Fe, Zn, Se and Cu. With age, regardless of the type of fat tissue, fat content decreased and water content increased. The highest content of protein, ash, cholesterol, some fatty acids (C18:0, C18:1n9c, C18:2n6c), generally higher content of MUFA, PUFA and the elements K, P, Ca, Mg, Fe, Zn, Pb, Se, Cr, Cd, were found in the fatty tissue of 15-year-old emus. Sex did influence the content of Si, Ca, Cu, Sr, which was higher in the fatty tissue of males. The composition of emu storage fat is determined by factors such as age, sex and the location of the fat tissue in the body.

## Introduction

The emu (*Dromaius novaehollandiae*) is world’s second largest bird and the largest avian species native to Australia. Its commercial farming has been continued since 1970. The species demonstrates a strong adaptability to diverse climatic conditions, as evidenced by its present range, covering almost every continent^1^. For example, there are more than one million emus farmed in the United States, while breeding of these birds in India has expanded to about 3,000 farms in over 15 states, which accounts for about 2.5 million birds^2^. The economic importance and increasing popularity of emu in various countries in the world is related to the versatile use of these birds. A number of valued materials are produced as a result of the emu farming, primarily meat, eggs, hide, and fat^1^.

Emu fat is the raw material used to produce valuable oil. These birds accumulate fat mainly under the skin and in the abdominal cavity. According to Birkbec^3^, depending on age, gender and condition, 4 to 15 kg of fat can be obtained from one emu. According to Lewis^4^, from a 50-week old emu about 4 kg of fat can be received, and from a 70-week old emu about 5 kg. Aborigines and early colonizers in Australia used emu fat to rub into wounds, to accelerate healing and relieve pain in inflammation^5^ and to soften and moisturize dry skin^6^.

In Australia, Canada, USA and Western Europe, oil-based pharmaceuticals are patented. The production of cosmetics based on this raw material is also popular^7^.

Its anti-inflammatory properties have been proven in various diseases. If applied topically, it reduces inflammation of the ears and inhibits progressive joint changes^8^. It contributes to the reduction of tumour necrosis factor and other cytokines in inflammatory and immunological responses^9^. It has antioxidant and free-radical absorption properties^10,11^. Administered orally, it is used to treat gastrointestinal inflammation^12–14^ and chemotherapy-induced bone problems^15^. Apart from anti-inflammatory properties, it shows hypocholesterolemic and antiatherosclerotic activity^16,17^. It stimulates skin renewal and hair growth, reduces wrinkles and rejuvenates the skin. It is also recommended for the treatment of discolorations and other disorders such as male pattern baldness and chemotherapy-induced alopecia^18^. It has good moisturizing and cosmetic properties^19^. It increases transdermal penetration, which can be used for transdermal administration of drugs and other substances^20–24^. It is an excellent insect repellent^25^. It is also used to treat psoriasis, burns and wounds^26,27^. It is believed that this oil has a number of other pro-health properties, among others, it is suggested that it may also be important in the treatment of diabetes^7^.

In poultry production, the raw material is obtained not only from young birds raised for slaughter, but also from older birds on the completion of the laying period. Raw material from such birds must also find its utility. Emu breeding herds are used for several laying season, which is due to the laying persistence of the birds. The literature lacks information on the quality of fat obtained from old emus after the end of their reproductive life. Apart from age, sex of the animal, genotype, feed composition and place of tissue deposition also affect fat composition^28,29,30,31^. Given the above, the aims of the research were:

- Determination of basic chemical composition, mineral profile and fatty acid profile in back fat and abdominal fat of 1, 3 and 15 year old male emu, and
- Determination of the impact of sex and location of adipose tissue in the body on the basic chemical composition, mineral profile and fatty acid profile of 15-year-old emus.

## Materials and methods

Two types of emu fat tissue (back fat and abdominal fat) were collected from a total of 26 birds aged 1 year (6 males), 3 years (6 males) and 15 years (8 females and 6 males) slaughtered in August. The experiment was carried out on an experimental farm of the Department of Utility and Decorative Birds Husbandry, West Pomeranian University of Technology in Szczecin.

Until the slaughter, the emu were kept in an open system, i.e. an open-air run with free access to grassy areas, regardless of weather conditions and season. All birds were fed the same standard compound feed in the form of granules based on wheat, barley, maize and soya pellets according to the dietary recommendations for this species. The compound feed contained 18% total protein, 6.7% crude ash, 5.2% crude fibre, 2.1% crude fat and 10.63 net metabolic energy in 1 kg of the feed. The birds were fed ad libitum. Before the slaughter, the birds did not receive food for 24 h.

The emu were killed by decapitation after being stunned. The birds were stunned by means of impact on the head with a wooden stick. After bleeding, stripping and skinning, samples of back fat and abdominal fat were collected from each bird. Fat samples were packed in twis-off plastic tubes, frozen and stored at − 80 °C until analyses were performed.

All procedures were performed according to the guidelines for the care and use of research animals and were approved by the Local Ethics Committee on Animal Experimentation in Szczecin (ZUT Szczecin) (resolution number 12/2014, Szczecin, PL).

### Proximate composition

The proximate composition of the fat was assessed by determining the percentage of basic chemical components (total water, total protein, fat and ash) using conventional methods^32^.

The cholesterol level, fatty acid profile and minerals content we determined using the methodology given by Bucław et al.^33^.

### Cholesterol level

The extracts obtained for determining the cholesterol content in the examined fats are described in the section Fatty acids content and profile (see text below). As with fatty acids, cholesterol was determined by the gas chromatography-mass spectrometry (GC–MS) method. The next steps of sample preparation and appropriate chromatographic analyses were made in accordance with the procedure described by Cunha et al.^34^

GC parameters for cholesterol: Capillary column COL-ELITE-5MS 30 m × 0.25 mm × 0.25 μm; carrier gas helium (He) 6.0; gas flow rate 1 ml/min; injection volume 1 μl; split injection ratio (split) 200:1; injector temperature 280 °C; p column temperature programme 100 °C for 5 min; temperature gradient 20 °C/min to 280 °C, 280 °C for 25 min; transfer line temperature 280 °C.

MS parameters for cholesterol: Selected Ion Recording (SIR) analysis: m/c = 329, ionisation energy 70 eV; ion source temperature 200 °C.

### Fatty acids profile

GC–MS was used to determine both the level of cholesterol and the profile of saturated fatty acids (SFAs), monounsaturated fatty acids (MUFAs) and polyunsaturated fatty acids (PUFAs).

Extraction: Before lipid extraction, all fats of a single bird specimen were placed in a polypropylene container and then, for easy homogenisation, a small, specific volume of demineralised water was added. Homogenisation in a laboratory blender was performed until the obtained mass showed complete homogeneity thereby providing the averaged samples to be collected for analysis. Thereafter, 2 g of homogenate taken from a number of points were placed in amber glass screw-capped vials (7.5 ml) with Teflon seal. Into each vial, 4 ml of chloroform were added and nitrogen was introduced. Then they were closed under continuous stream of nitrogen and vigorously shaken for 2 h. In order to separate the chloroform phase from the non-lipid residue, the vials were centrifuged at 2000 rpm for 20 min. The extraction process was repeated three more times to elute all the lipids completely, and extracts were combined together and filled up with chloroform to 20 ml.

Hydrolysis: The chloroform phase was placed into amber glass vials (4 ml) in an amount corresponding to 5 mg of extracted lipids and chloroform was evaporated under a stream of nitrogen. The vials were then closed with a screw cap equipped with a vent allowing neutral gas and reagents to be introduced without air entering. The vials were immediately filled up with nitrogen, and 400 μl of 0.5 M KOH in methanol solution were added and incubated in a heating block at 80 °C for 20 min.

Esterification: After cooling, 500 μl of 14% boron trifluoride (BF3) in methanol solution were introduced into each vial and incubated at 80 °C for 35 min. In order to extract fatty acid methyl esters (FAME), 1 ml of saturated NaCl solution and 2 ml of isooctane were added to each cooled vial as an extractant, vigorously shaken for 1 h, and left for 0.5 h until the phases separated. The upper isooctane layer was collected to separate vials containing about 0.6 g of anhydrous sodium sulphate (Na2SO4). The vials were then filled with nitrogen and left for 2 h. The dried FAME extracts were placed in vials into an automatic sample changer (autosampler) of the gas chromatograph.

GC–MS: The determination of fatty acid methyl esters in liver lipids was made by the GC–MS method on a PerkinElmer CLARUS^®^ 600 GC/MS with a capillar y column COL-ELITE-5MS 60 m × 0.25 mm × 0.25 μm. A Supelco 37 component mixture (F.A.M.E. Mix C4–C24) was the fatty acid standard.

GC parameters: Carrier gas helium (He) 6.0; gas flow rate 1 ml/min; injection volume 1 μl; split injection ratio 50:1; injector temperature 200 °C; column temperature programme 110 °C for 5 min; temperature gradient 5 °C/min to 180 °C, 180 °C for 15 min; temperature gradient 5 °C/min to 290 °C, 290 °C for 5 min; transfer line temperature 290 °C.

MS parameters: SIR analysis according to selected mass/charge (m/c) abundances; ionisation energy 70 eV; ion source temperature 200 °C; selected ions and retention time (Table 1).

**Table 1:** Selected abundances (m/z*) and retention time (RT) for fatty acid methyl ester (FAME) GC MS analysis.

**m/z**	**FAME (RT—minute)**
55	C14:1 (33.72), C15:1 (39.37), C16:1 (43.03), C17:1 (46.26), C18:1n9c (48.93), C20:1 (53.32), C22:1n9 (56.96), C24:1 (60.28)
67	C20:3n6 (52.22), C22:2 (56,86)
74	C8:0 (12.76), C10:0 (18.21), C11:0 (21.04), C12:0 (24.32), C13:0 (28.60), C14:0 (34.51), C16:0 (43.79), C17:0 (46.93), C18:0 (49.52), C20:0 (53.80), C21:0 (55.64), C22:0 (57.32), C23:0 (58.96), C24:0 (60.66)
79	C20:4n6 (52.48), C20:5n3 (52.62)
194	C18:3n6 (48.32), C22:6n3 (56.12)
292	C18:3n3 (48.75)
294	C18:2n6c (48.75)
296	C18:1n9t (49,05)

*Ion mass to charge ratio.

### Minerals content

The levels of minerals in fats were determined by inductively coupled plasma optical emission spectrometry using the Optima 2000 DV ICP-AES (PerkinElmer Inc., Germany) following digestion in a microwave oven type Multiwave (Anton Paar GmbH, Austria) equipped with a system of continuous temperature and pressure control in each quartz vessel. The weighed amount of tissue homogenate (ca. 1 g) was transferred to a pressure quartz glass vessel, into which 5.0 ml of 65% HNO_3_ and 0.5 ml of 30% H_2_O_2_ (both Suprapur, Merck) were successively added. Mineralisation was conducted according to the equipment application mode MEAT: 0–5 min—linear gradient of power 100–600 W; 6–10 min—600 W (constant); 11–20 min—1,000 W or less after reaching 75 MPa or 300 °C; 21–35 min—vessel cooling. The cooled and degassed mineralisate was filled up to 10 ml in volumetric flasks.

Microelements (As, Ba, Cd, Cr, Cu, Fe, Mn, Se, Si, Sr, Pb and Zn) were directly determined in the solutions prepared this way, whereas mineralisates were diluted 10- or 100-fold for the determination of macroelements (Ca, Mg, Na, K and P) in order to obtain the range of linear dependence of emission signal on the concentration of a given mineral component. Measurements of the intensity of emitted radiation for micro-elements were made selecting a longer, axial optical path (along plasma), whereas macroelements were analysed radially (across plasma). As standard, a certified multi-element solution for ICP (ICP Multielement Standard IV, Merck) was used. Standard solutions were supplemented with the addition of acid used in mineralisation, in the concentration which occurred in mineralised samples. In order to minimise potential interferences in sample introduction to plasma and other physical disturbances, analyses were made using the internal standard method by yttrium (Y) introduction into sample and standard solutions in a concentration of 0.5 mg Y/l.

### Statistical analyses

The results were statistically analysed using the Statistica 13.1 PL software package (IBM Corp.; SPSS Statistics for Windows; Version 23.0; 2016). Two-way ANOVA was used to determine if two different factors: fat type and emu age and fat type and emu sex have an effect on a measured variable. Least Squares Means were obtained using the Tukey test. The significance was calculated at a 5% confidence level.

### Ethics approval

All procedures were performed according to the guidelines for the care and use of research animals and were approved by the Local Ethics Committee on Animal Experimentation in Szczecin (ZUT Szczecin) (resolution number 12/2014, Szczecin, PL).

### Consent for publication

All authors they express their consent for publication.

## Results

### Proximate composition

When analysing the basic chemical composition of back fat and abdominal fat in emu male depending on the age (Table 2), it was noted that the place of fat deposition influenced the content of protein, fat and ash (*P ≤ *0.05). More than twice as much protein content was found in back fat (1.13%). Abdominal fat was characterized by a higher content of fat (96.6%) and ash (0.081%). The content of all the studied basic components depended on the age of emu (*P ≤ *0.05). With age, the amount of water in the fatty tissue increased, regardless of its type, with decreasing fat content. These changes were linear and concerned the 1-year, 3-year and 15-year-old male emus. Fat of the 15-year-old male emus was characterized by a higher proportion of water (2.66%), protein (1.14%) and ash (0.081%), and a lower presence of fat (95.7%). Significant interactions (type of fat × age of emu) were found for water and fat content (*P ≤ *0.05). The highest water content was recorded in fat of the 15-year-old male birds, especially in abdominal fat (2.74%). In comparison, abdominal fat of the 1-yer-old emus contained 1.02% water. Also in this fat a higher level of fat was detected (98.0%), while its lowest share was in fat from the 15-year-old male emus (95.7%).

**Table 2:** Basic chemical composition (%) of back fat and abdominal fat of emu males depending on age (mean ± SE).

**Item**	**Fat type**	**Emu age**				**Main effect**		
		**1-year old** **n = 6**	**3-years old** **n = 6**	**15-years old** **n = 6**	**Average fat type**	**Type**	**Age**	**Type × Age**
Water	Back fat	1.81^c^ ± 0.10	1.99^bc^ ± 0.13	2.58^ab^ ± 0.17	2.13 ± 0.11	0.0673	< 0.0001	0.0042
	Abdominal fat	1.02^d^ ± 0.14	1.98^c^ ± 0.14	2.74^a^ ± 0.15	1.91 ± 0.19			
	Average emu age	1.42_c_ ± 0.14	1.99_b_ ± 0.09	2.66_a_ ± 0.11	2.02 ± 0.11			
Protein	Back fat	0.93 ± 0.06	1.03 ± 0.06	1.42 ± 0.07	1.13* ± 0.06	< 0.0001	< 0.0001	0.5929
	Abdominal fat	0.33 ± 0.02	0.39 ± 0.02	0.87 ± 0.03	0.53* ± 0.06			
	Average emu age	0.63_b_ ± 0.09	0.71_b_ ± 0.10	1.14_a_ ± 0.09	0.83 ± 0.07			
Lipids	Back fat	96.8^b^ ± 0.1	96.6^b^ ± 0.2	95.7^c^ ± 0.2	96.4* ± 0.1	0.0004	< 0.0001	0.0056
	Abdominal fat	98.0^a^ ± 0.2	97.0^b^ ± 0.1	95.7^c^ ± 0.2	96.9* ± 0.2			
	Average emu age	97.4_a_ ± 0.1	96.8_b_ ± 0.1	95.7_c_ ± 0.1	96.6 ± 0.1			
Ash	Back fat	0.072 ± 0.001	0.073 ± 0.002	0.076 ± 0.003	0.074* ± 0.001	< 0.0001	0.0018	0.1554
	Abdominal fat	0.076 ± 0.001	0.080 ± 0.002	0.086 ± 0.002	0.081* ± 0.001			
	Average emu age	0.074^b^ ± 0.001	0.077_b_ ± 0.001	0.081_a_ ± 0.002	0.077 ± 0.001			

*The same symbol in the superscript indicates statistically significant differences (P ≤ 0.05) for the type of fat.

_abc_Statistically significant differences (P ≤ 0.05) for the emu age were marked with different letters in the subscript.

^abc^Statistically significant differences (P ≤ 0.05) for interaction type of fat × age of emu were marked with different letters in the upper index.

The analysis of the 15-year-old female and male emus’ adipose tissue (Table 3) revealed the influence of its location in the emu body upon protein, fat and ash content (*P ≤ *0.05). Back fat was characterized by a higher protein content (1.44%) while abdominal fat that of fat (95.9%) and ash (0.083%). No influence of gender and interaction fat type × gender on the basic chemical composition was observed in 15-year-old female and male emus (*P ≥ *0.05).

**Table 3:** Basic chemical composition (%) of back fat and abdominal fat of 15-years-old emu depending on sex (mean ± SE).

**Item**	**Fat type**	**Emu sex**			**Main effect**		
		**Female** **n = 8**	**Male** **n = 6**	**Average fat type**	**Type**	**Sex**	**Type × Sex**
Water	Back fat	3.03 ± 0.19	2.58 ± 0.17	2.84 ± 0.14	0.7496	0.1897	0.2330
	Abdominal fat	2.76 ± 0.17	2.74 ± 0.15	2.75 ± 0.11			
	Average emu sex	2.89 ± 0.13	2.66 ± 0.11	2.79 ± 0.09			
Protein	Back fat	1.46 ± 0.06	1.42 ± 0.07	1.44* ± 0.04	< 0.0001	0.8270	0.4686
	Abdominal fat	0.84 ± 0.03	0.87 ± 0.03	0.85* ± 0.02			
	Average emu sex	1.15 ± 0.09	1.14 ± 0.09	1.15 ± 0.09			
Lipids	Back fat	95.3 ± 0.2	95.7 ± 0.2	95.5* ± 0.2	0.0297	0.9131	0.0528
	Abdominal fat	96.1 ± 0.2	95.7 ± 0.2	95.9* ± 0.1			
	Average emu sex	95.7 ± 0.2	95.7 ± 0.1	95.7 ± 0.1			
Ash	Back fat	0.075 ± 0.001	0.076 ± 0.003	0.075* ± 0.001	< 0.0001	0.0675	0.1253
	Abdominal fat	0.081 ± 0.001	0.086 ± 0.002	0.083* ± 0.001			
	Average emu sex	0.078 ± 0.001	0.081 ± 0.002	0.079 ± 0.001			

*The same symbol in the superscript indicates statistically significant differences (P ≤ 0.05) for the type of fat.

_abc_Statistically significant differences (P ≤ 0.05) for the emu age were marked with different letters in the subscript.

^abc^Statistically significant differences (P ≤ 0.05) for interaction type of fat × age of emu were marked with different letters in the upper index.

### Cholesterol level

A significant effect of emu male age (*P ≤ *0.05) and localization of adipose tissue (*P ≤ *0.05) on cholesterol content (Table 4) was found. Its higher content was found in back fat (61.3 mg/100 g). The highest proportion of cholesterol was found in fat of 15-year-old male emus (63.4 mg/100 g).

**Table 4:** Cholesterol content (mg/100 g) and fatty acid profile (% of total fatty acid) of back fat and abdominal fat of emu males depending on age (mean ± SE).

**Item**	**Fat type**	**Emu age**				**Main effect**		
		**1-year old** **n = 6**	**3-years old** **n = 6**	**15-years old** **n = 6**	**Average fat type**	**Type**	**Age**	**Type × Age**
Cholesterol	Back fat	58.7 ± 1.0	59.8 ± 1.3	65.4 ± 1.1	61.3* ± 0.9	0.0083	0.0002	0.0548
	Abdominal fat	59.3 ± 1.2	53.2 ± 1.8	61.3 ± 2.0	58.0* ± 1.3			
	Average emu age	59.0_b_ ± 0.8	56.5_b_ ± 1.5	63.4_a_ ± 1.2	59.7 ± 0.8			
C16:0	Back fat	41.6 ± 0.5	41.4 ± 0.6	26.9 ± 0.8	36.7 ± 1.7	0.2761	< 0.0001	0.5991
	Abdominal fat	41.8 ± 0.5	40.4 ± 0.8	25.8 ± 0.9	36.0 ± 1.8			
	Average emu age	41.7_a_ ± 0.4	40.9_a_ ± 0.5	26.3_b_ ± 0.6	36.3 ± 1.2			
C18:0	Back fat	11.7 ± 0.4	12.6 ± 0.2	17.0 ± 1.2	13.8 ± 0.7	0.2739	< 0.0001	0.9840
	Abdominal fat	12.2 ± 0.1	13.1 ± 0.2	17.7 ± 0.8	14.3 ± 0.6			
	Average emu age	11.9_b_ ± 0.2	12.8_b_ ± 0.1	17.3_a_ ± 0.7	14.0 ± 0.5			
Other SFA	Back fat	0.63 ± 0.02	0.58 ± 0.02	0.28 ± 0.02	0.50 ± 0.04	0.1564	< 0.0001	0.2252
	Abdominal fat	0.64 ± 0.02	0.58 ± 0.03	0.36 ± 0.03	0.52 ± 0.03			
	Average emu age	0.63_a_ ± 0.01	0.58_a_ ± 0.02	0.32_b_ ± 0.02	0.51 ± 0.02			
SFA	Back fat	54.0 ± 0.5	54.6 ± 0.7	44.2 ± 1.6	50.9 ± 1.3	0.9361	< 0.0001	0.7752
	Abdominal fat	54.7 ± 0.5	54.1 ± 0.9	43.8 ± 0.9	50.9 ± 1.3			
	Average emu age	54.3_a_ ± 0.3	54.4_a_ ± 0.5	44.0_b_ ± 0.9	50.9 ± 0.9			
C16:1	Back fat	4.82 ± 0.34	5.28 ± 0.15	3.52 ± 0.25	4.54* ± 0.23	0.0260	< 0.0001	0.8628
	Abdominal fat	4.49 ± 0.31	4.70 ± 0.22	2.93 ± 0.27	4.04* ± 0.24			
	Average emu age	4.66_a_ ± 0.22	4.99_a_ ± 0.15	3.23_b_ ± 0.19	4.29 ± 0.17			
C18:1n9c	Back fat	23.4 ± 0.6	24.5 ± 0.6	34.1 ± 0.8	27.3 ± 1.2	0.6039	< 0.0001	0.8347
	Abdominal fat	23.2 ± 0.7	25.3 ± 0.8	34.5 ± 1.2	27.7 ± 1.3			
	Average emu age	23.3_b_ ± 0.5	24.9_b_ ± 0.5	34.3_a_ ± 0.7	27.5 ± 0.9			
C18:1n9t	Back fat	3.63 ± 0.08	3.96 ± 0.20	2.94 ± 0.18	3.51 ± 0.14	0.9127	< 0.0001	0.6261
	Abdominal fat	3.57 ± 0.14	3.87 ± 0.21	3.14 ± 0.14	3.52 ± 0.12			
	Average emu age	3.60_a_ ± 0.08	3.91_a_ ± 0.14	3.04_b_ ± 0.11	3.52 ± 0.09			
Other MUFA	Back fat	0.60^ab^ ± 0.01	0.61^ab^ ± 0.01	0.23^c^ ± 0.01	0.48* ± 0.04	0.0334	< 0.0001	0.0219
	Abdominal fat	0.57^b^ ± 0.01	0.66^a^ ± 0.03	0.30^c^ ± 0.02	0.51* ± 0.04			
	Average emu age	0.59_b_ ± 0.01	0.64_a_ ± 0.02	0.27_c_ ± 0.02	0.50 ± 0.03			
MUFA	Back fat	32.4 ± 0.5	34.4 ± 0.7	40.8 ± 0.8	35.9 ± 0.9	0.8642	< 0.0001	0.8677
	Abdominal fat	31.9 ± 0.6	34.6 ± 0.8	40.8 ± 1.0	35.8 ± 1.0			
	Average emu age	32.2_c_ ± 0.4	34.5_b_ ± 0.5	40.8_a_ ± 0.6	35.8 ± 0.7			
C18:2n6c	Back fat	12.3 ± 0.2	9.9 ± 0.1	14.2 ± 1.0	12.1 ± 0.5	0.5956	< 0.0001	0.8730
	Abdominal fat	12.2 ± 0.2	10.2 ± 0.1	14.6 ± 0.7	12.3 ± 0.5			
	Average emu age	12.3_b_ ± 0.1	10.1_c_ ± 0.1	14.4_a_ ± 0.6	12.2 ± 0.4			
C18:3n3	Back fat	0.86 ± 0.01	0.69 ± 0.01	0.70 ± 0.11	0.75 ± 0.04	0.4063	0.0025	0.3553
	Abdominal fat	0.86 ± 0.02	0.72 ± 0.01	0.56 ± 0.10	0.71 ± 0.04			
	Average emu age	0.86_a_ ± 0.01	0.70_b_ ± 0.01	0.63_b_ ± 0.07	0.73 ± 0.03			
Other PUFA	Back fat	0.43 ± 0.03	0.43 ± 0.02	0.16 ± 0.02	0.34 ± 0.03	0.4307	< 0.0001	0.2021
	Abdominal fat	0.38 ± 0.03	0.40 ± 0.01	0.19 ± 0.03	0.32 ± 0.03			
	Average emu age	0.41_a_ ± 0.02	0.42_a_ ± 0.01	0.17_b_ ± 0.02	0.33 ± 0.02			
PUFA	Back fat	13.6 ± 0.2	11.0 ± 0.1	15.0 ± 1.0	13.2 ± 0.5	0.7108	< 0.0001	0.8899
	Abdominal fat	13.5 ± 0.3	11.3 ± 0.1	15.3 ± 0.8	13.4 ± 0.5			
	Average emu age	13.5_b_ ± 0.2	11.2_c_ ± 0.1	15.2_a_ ± 0.6	13.3 ± 0.3			

*The same symbol in the superscript indicates statistically significant differences (P ≤ 0.05) for the type of fat.

_abc_Statistically significant differences (P ≤ 0.05) for the emu age were marked with different letters in the subscript.

^abc^Statistically significant differences (P ≤ 0.05) for interaction type of fat × age of emu were marked with different letters in the upper index.

Other SFA = C8:0 + C10:0 + C11:0 + C11:0 + C12:0 + C13:0 + C14:0 + C15:0 + C17:0 + C20:0 + C21:0 + C22:0 + C23:0 + C24:0

SFA = C16:0 + C18:0 + Other SFA.

Other MUFA = C14:1 + C15:1 + C17:1 + C20:1 = C22:1n9 + C24:1

MUFA = C16:1 + C18:1n9c + C18:1n9t + Other MUFA.

Other PUFA = C18:3n6 + C20:3n3 + C20:3n6 + C20:4n6 + C20:5n3 + C22:2 + C22:6n3.

PUFA = C18:2n6c + C18:3n3 + Other PUFA.

Comparing the cholesterol content in fat of the 15-year-old females and males of emu (Table 5), only a significant effect of the type of fat was observed (*P ≤ *0.05). The amount of cholesterol in back fat was higher by an average of 3.4 mg/100 g compared to abdominal fat (62.1 mg/100 g).

**Table 5:** Cholesterol content (mg/100 g) and fatty acid profile (% of total fatty acid) of back fat and abdominal fat of 15-years-old emu depending on sex (mean ± SE).

**Item**	**Fat type**	**Emu sex**			**Main effect**		
		**Female** **n = 8**	**Male** **n = 6**	**Average fat type**	**Type**	**Sex**	**Type × Sex**
Cholesterol	Back fat	65.6 ± 1.5	65.4 ± 1.1	65.5* ± 0.09	0.0253	0.6041	0.6699
	Abdominal fat	62.7 ± 1.1	61.3 ± 2.0	62.1* ± 1.0			
	Average emu sex	64.2 ± 1.0	63.4 ± 1.2	63.8 ± 0.8			
C16:0	Back fat	28.3 ± 1.3	26.9 ± 0.8	27.7 ± 0.8	0.4303	0.2116	0.9192
	Abdominal fat	27.5 ± 1.4	25.8 ± 0.9	26.7 ± 0.9			
	Average emu sex	27.9 ± 0.9	26.3 ± 0.6	27.2 ± 0.6			
C18:0	Back fat	15.3 ± 0.6	17.0 ± 1.2	16.0 ± 0.6	0.1853	0.0934	0.6415
	Abdominal fat	16.7 ± 0.6	17.7 ± 0.8	17.1 ± 0.5			
	Average emu sex	16.0 ± 0.4	17.3 ± 0.7	16.6 ± 0.4			
Other SFA	Back fat	0.29 ± 0.01	0.28 ± 0.02	0.29* ± 0.01	0.0049	0.3727	0.2389
	Abdominal fat	0.32 ± 0.01	0.36 ± 0.03	0.34* ± 0.01			
	Average emu sex	0.31 ± 0.01	0.32 ± 0.02	0.31 ± 0.01			
SFA	Back fat	43.9 ± 1.6	44.2 ± 1.6	44.0 ± 1.1	0.9480	0.8855	0.7743
	Abdominal fat	44.5 ± 1.7	43.8 ± 0.9	44.2 ± 1.0			
	Average emu sex	44.2 ± 1.1	44.0 ± 0.9	44.1 ± 0.7			
C16:1	Back fat	4.13 ± 0.31	3.52 ± 0.25	3.87* ± 0.21	0.0276	0.0842	0.7607
	Abdominal fat	3.36 ± 0.29	2.93 ± 0.27	3.18* ± 0.20			
	Average emu sex	3.75 ± 0.23	3.23 ± 0.19	3.52 ± 0.16			
C18:1n9c	Back fat	33.6 ± 0.7	34.1 ± 0.8	33.8 ± 0.5	0.9071	0.4258	0.7972
	Abdominal fat	33.5 ± 1.0	34.5 ± 1.2	33.9 ± 0.7			
	Average emu sex	33.5 ± 0.6	34.3 ± 0.7	33.9 ± 0.4			
C18:1n9t	Back fat	2.94 ± 0.14	2.94 ± 0.18	2.94 ± 0.11	0.3227	0.7684	0.7678
	Abdominal fat	3.05 ± 0.14	3.14 ± 0.14	3.08 ± 0.10			
	Average emu sex	2.99 ± 0.10	3.04 ± 0.11	3.01 ± 0.07			
Other MUFA	Back fat	0.22 ± 0.03	0.23 ± 0.01	0.22 ± 0.02	0.1425	0.3984	0.6098
	Abdominal fat	0.25 ± 0.04	0.30 ± 0.02	0.27 ± 0.03			
	Average emu sex	0.24 ± 0.03	0.27 ± 0.02	0.25 ± 0.02			
MUFA	Back fat	40.9 ± 0.8	40.8 ± 0.8	40,.9 ± 0.5	0.7002	0.7406	0.6753
	Abdominal fat	40.1 ± 1.0	40.8 ± 1.0	40.4 ± 0.7			
	Average emu sex	40.5 ± 0.6	40.8 ± 0.6	40.7 ± 0.4			
C18:2n6c	Back fat	14.1 ± 1.1	14.2 ± 1.0	14.1 ± 0.7	0.7200	0.9004	0.9482
	Abdominal fat	14.4 ± 1.0	14.6 ± 0.7	14.5 ± 0.6			
	Average emu sex	14.3 ± 0.7	14.4 ± 0.6	14.3 ± 0.5			
C18:3n3	Back fat	0.84 ± 0.14	0.70 ± 0.11	0.78 ± 0.09	0.3041	0.1934	0.8812
	Abdominal fat	0.74 ± 0.10	0.56 ± 0.10	0.66 ± 0.07			
	Average emu sex	0.79 ± 0.08	0.63 ± 0.07	0.72 ± 0.06			
Other PUFA	Back fat	0.22 ± 0.05	0.16 ± 0.02	0.19 ± 0.03	0.6284	0.1960	0.6309
	Abdominal fat	0.22 ± 0.03	0.19 ± 0.03	0.21 ± 0.02			
	Average emu sex	0.22 ± 0.03	0.17 ± 0.02	0.20 ± 0.02			
PUFA	Back fat	15.2 ± 1.3	15.0 ± 1.0	15.1 ± 0.8	0.8195	0.9432	0.9534
	Abdominal fat	15.4 ± 1.1	15.3 ± 0.8	15.4 ± 0.7			
	Average emu sex	15.3 ± 0.8	15.2 ± 0.6	15.2 ± 0.5			

*The same symbol in the superscript indicates statistically significant differences (P ≤ 0.05) for the type of fat.

_abc_Statistically significant differences (P ≤ 0.05) for the emu age were marked with different letters in the subscript.

^abc^Statistically significant differences (P ≤ 0.05) for interaction type of fat × age of emu were marked with different letters in the upper index.

Other SFA = C8:0 + C10:0 + C11:0 + C11:0 = C12:0 + C13:0 + C14:0 + C15:0 + C17:0 + C20:0 + C21:0 + C22:0 + C23:0 + C24:0

SFA = C16:0 + C18:0 + Other SFA.

Other MUFA = C14:1 + C15:1 + C17:1 + C20:1 = C22:1n9 + C24:1

MUFA = C16:1 + C18:1n9c + C18:1n9t + Other MUFA. Other PUFA = C18:3n6 + C20:3n3 + C20:3n6 + C20:4n6 + C20:5n3 + C22:2 + C22:6n3.

PUFA = C18:2n6c + C18:3n3 + Other PUFA.

### Fatty acids profiles

The analysis of back fat and abdominal fat of male emus at different ages (Table 4) revealed the effect of localization of fat tissue on the proportion of palmitoleic acid (C16:1; *P ≤ *0.05). Back fat was characterized by its higher content (4.54%). Differences were also found in other monounsaturated fatty acids (other MUFA; *P ≤ *0.05), whose greater share was recorded in abdominal fat (0.51%). The profile of fatty acids was dependent on the age of emu (*P ≤ *0.05). It was found that fat of the 15-year-old male emus was characterized by a higher proportion of stearic acid (C18:0; 17.3%), *cis* oleic acid (C18:1n9c; 34.3%) and linoleic acid (C18:2n6c; 12.2%), monounsaturated fatty acids (MUFA; 40.8%) and polyunsaturated fatty acids (PUFA; 15.2%). A smaller share was observed for palmitic acid (C16:0; 26.3%), C16:1 (3.23%), *trans* oleic acid (C18:1n9t; 3.04%) and other fatty acids (Other SFA—0.32%, Other MUFA—0.27%, Other PUFA—0.17%) and for saturated acids (SFA; 44.0%) in comparison to the 1-year and 3-year-old male emus. A smaller share of α -linolenic acid (C18:3n3; 3.04%) was also recorded in comparison to 1-year-old birds. A significant interaction (fat type × age of emu) was only found in case of other MUFA (*P ≤ 0*.05).

No gender or fat type × emu age interaction (*P ≥ 0*.05) was found to affect the fatty acid profile of 15-year-old female and male emu fat (Table 5). The effect of fat type was proved in case of other SFA (*P ≤ 0*.05; back fat − 0.29%, depot fat − 0.34%) and C16:1 (*P ≤ 0*.05), where its greater share was found in back fat (3.87%).

### Minerals content

It was found that the type of fat did not affect the content of Pb, Cr, Cd and Sr (*P ≥ *0.05; Table 6). For other elements, except Ba and Mn, back fat contained more of them than abdominal fat (*P ≤ *0.05). The age of emu male affected the content of all studied elements (*P ≤ *0.05). Fat of 15-year-old male emus contained the most K (128.0 mg/kg), P (95.1 mg/kg), Ca (15.1 mg/kg), Mg (6.78 mg/kg), Fe (3.18 mg/kg), Zn (1.10 mg/kg), Pb (0.198 mg/kg), Se (103 mg/kg), Cr (0.0444 mg/kg), Cd (0.0215 mg/kg) and Sr (0.0206 mg/kg) and the least Si (74.1 mg/kg), Na (33.4 mg/kg) and Ba (0.050 mg/kg) compared to that of the younger birds. Fat of 1-year-old male emus was characterized by the highest content of Mn (0.0109 mg/kg) and the lowest content of Cu (0.057 mg/kg) in comparison to the older birds. Significant interactions (fat type × age of emu) were found in the content of K, Si, Ca, Fe, Se, Cu and Mn (*P ≤ *0.05). The highest levels of K (145.5 mg/kg), Ca (18.8 mg/kg), Fe (4.09 mg/kg), Se (0.115 mg/kg) are characteristic for dorsal fat of the 15-year-old male emus, Si (185.5 mg/kg) for dorsal fat of the 3-year-old male emus and Mn (0.0204 mg/kg) for abdominal fat of the 1-year-old male emus.

**Table 6:** Mineral content (mg/kg) of back fat and abdominal fat of emu males depending on age (mean ± SE).

**Item**	**Fat type**	**Emu age**				**Main effect**		
		**1-year old** **n = 6**	**3-years old** **n = 6**	**15-years old** **n = 6**	**Average type fat**	**Type**	**Age**	**Type × Age**
Na	Back fat	74.4 ± 5.1	64.3 ± 5.0	45.6 ± 2.7	61.4* ± 3.8	< 0.0001	< 0.0001	0.8844
	Abdominal fat	48.7 ± 4.3	42.4 ± 3.2	21.2 ± 2.1	37.4* ± 3.4			
	Average emu age	61.5_a_ ± 5.0	53.4_a_ ± 4.4	33.4_b_ ± 4.0	49.4 ± 3.2			
K	Back fat	67.2^c^ ± 3.1	59.2^ cd^ ± 2.4	145.5^a^ ± 7.1	90.6* ± 9.8	< 0.0001	< 0.0001	0.0134
	Abdominal fat	45.9^d^ ± 2.9	49.9^ cd^ ± 1.3	110.6^b^ ± 4.8	68.8* ± 7.4			
	Average emu age	56.6_b_ ± 3.8	54.5_b_ ± 1.9	128.0_a_ ± 6.7	79.7 ± 6.3			
Ca	Back fat	13.7^b^ ± 0.8	13.5^b^ ± 0.5	18.8^a^ ± 0.7	15.3* ± 0.7	< 0.0001	0.0003	< 0.0001
	Abdominal fat	12.3^b^ ± 0.7	11.4^b^ ± 0.4	11.5^b^ ± 0.4	11.7* ± 0.3			
	Average emu age	13.0_b_ ± 0.5	12.5_b_ ± 0.4	15.1_a_ ± 1.2	13.5 ± 0.5			
Mg	Back fat	5.14 ± 0.23	5.03 ± 0.17	7.68 ± 0.70	5.95* ± 0.38	0.0005	< 0.0001	0.2297
	Abdominal fat	4.50 ± 0.24	4.17 ± 0.10	5.89 ± 0.27	4.85* ± 0.22			
	Average emu age	4.82_b_ ± 0.18	4.60_b_ ± 0.16	6.78_a_ ± 0.45	5.40 ± 0.23			
P	Back fat	60.3 ± 2.1	55.9 ± 1.8	105.1 ± 5.0	73.8* ± 5.7	< 0.0001	< 0.0001	0.0947
	Abdominal fat	45.0 ± 2.1	47.5 ± 1.4	85.1 ± 1.0	59.2* ± 4.5			
	Average emu age	52.6_b_ ± 2.7	51.7_b_ ± 1.7	95.1_a_ ± 3.9	66.5 ± 3.8			
Fe	Back fat	1.49^bc^ ± 0.15	1.36^c^ ± 0.12	4.09^a^ ± 0.30	2.31* ± 0.32	0.0004	< 0.0001	< 0.0001
	Abdominal fat	1.55^bc^ ± 0.15	1.25^c^ ± 0.12	2.28^b^ ± 0.23	1.69* ± 0.14			
	Average emu age	1.52_b_ ± 0.10	1.30_b_ ± 0.08	3.18_a_ ± 0.33	2.00 ± 0.18			
Cr	Back fat	0.0101 ± 0.0001	0.0099 ± 0.0001	0.0438 ± 0.0031	0.0212 ± 0.0040	0.7783	< 0.0001	0.9127
	Abdominal fat	0.0102 ± 0.0002	0.0097 ± 0.0001	0.0450 ± 0.0030	0.0217 ± 0.0041			
	Average emu age	0.0101_b_ ± 0.0001	0.0098_b_ ± 0.0001	0.0444_a_ ± 0.0021	0.0214 ± 0.0028			
Cu	Back fat	0.045^d^ ± 0.003	0.168^a^ ± 0.016	0.118^b^ ± 0.014	0.111* ± 0.014	0.0003	< 0.0001	< 0.0001
	Abdominal fat	0.068^ cd^ ± 0.008	0.053^d^ ± 0.007	0.103^bc^ ± 0.011	0.075* ± 0.007			
	Average emu age	0.057_b_ ± 0.005	0.111_a_ ± 0.019	0.111_a_ ± 0.009	0.093 ± 0.008			
Mn	Back fat	0.0014^d^ ± 0.0002	0.0004^d^ ± 0.0001	0.0053^c^ ± 0.0004	0.0024* ± 0.0005	< 0.0001	< 0.0001	< 0.0001
	Abdominal fat	0.0204^a^ ± 0.0007	0.0098^b^ ± 0.0002	0.0044^c^ ± 0.0003	0.0115* ± 0.0016			
	Average emu age	0.0109_a_ ± 0.0029	0.0051_b_ ± 0.0014	0.0048_b_ ± 0.0003	0.0070 ± 0.0011			
Se	Back fat	0.063^c^ ± 0.005	0.068^c^ ± 0.003	0.115^a^ ± 0.004	0.082* ± 0.006	0.0031	< 0.0001	0.0048
	Abdominal fat	0.067^c^ ± 0.004	0.058^c^ ± 0.002	0.091^b^ ± 0.005	0.072* ± 0.004			
	Average emu age	0.065_b_ ± 0.003	0.063_b_ ± 0.002	0.103_a_ ± 0.005	0.077 ± 0.004			
Si	Back fat	123.2^b^ ± 6.0	185.5^a^ ± 4.8	77.6^c^ ± 2.7	128.8* ± 11.0	< 0.0001	< 0.0001	< 0.0001
	Abdominal fat	111.6^b^ ± 6.9	107.0^b^ ± 6.2	70.7^c^ ± 2.3	96.4* ± 5.4			
	Average emu age	117.4_b_ ± 4.7	146.2_a_ ± 12.4	74.1_c_ ± 2.0	112.6 ± 6.6			
Zn	Back fat	1.01 ± 0.06	0.85 ± 0.05	1.28 ± 0.11	1.05* ± 0.06	0.0015	0.0006	0.0693
	Abdominal fat	0.88 ± 0.06	0.79 ± 0.04	0.92 ± 0.04	0.86* ± 0.03			
	Average emu age	0.94_b_ ± 0.04	0.82_b_ ± 0.03	1.10_a_ ± 0.08	0.95 ± 0.04			
Ba	Back fat	0.121 ± 0.004	0.114 ± 0.008	0.050 ± 0.005	0.095* ± 0.008	0.0091	< 0.0001	0.1517
	Abdominal fat	0.138 ± 0.004	0.134 ± 0.005	0.049 ± 0.004	0.107* ± 0.010			
	Average emu age	0.130_a_ ± 0.004	0.124_a_ ± 0.005	0.050_b_ ± 0.003	0.101 ± 0.007			
Cd	Back fat	0.0039 ± 0.0001	0.0038 ± 0.0001	0.0211 ± 0.0021	0.0096 ± 0.0021	0.7369	< 0.0001	0.9249
	Abdominal fat	0.0039 ± 0.0001	0.0039 ± 0.0001	0.0219 ± 0.0014	0.0099 ± 0.0021			
	Average emu age	0.0039_b_ ± 0.0001	0.0039_b_ ± 0.0001	0.0215_a_ ± 0.0012	0.0097 ± 0.0015			
Pb	Back fat	0.034 ± 0.001	0.036 ± 0.001	0.199 ± 0.010	0.090 ± 0.019	0.9423	< 0.0001	0.9882
	Abdominal fat	0.035 ± 0.001	0.036 ± 0.001	0.198 ± 0.008	0.089 ± 0.019			
	Average emu age	0.035_b_ ± 0.001	0.036_b_ ± 0.001	0.198_a_ ± 0.006	0.090 ± 0.013			
Sr	Back fat	0.0016 ± 0.0001	0.0015 ± 0.0001	0.0207 ± 0.0020	0.0079 ± 0.0023	0.7997	< 0.0001	0.9924
	Abdominal fat	0.0012 ± 0.0001	0.0013 ± 0.0001	0.0206 ± 0.0016	0.0077 ± 0.0023			
	Average emu age	0.0014_b_ ± 0.0001	0.0014_b_ ± 0.0001	0.0206_a_ ± 0.0012	0.0078 ± 0.0016			

*The same symbol in the superscript indicates statistically significant differences (P ≤ 0.05) for the type of fat.

_abc_Statistically significant differences (P ≤ 0.05) for the emu age were marked with different letters in the subscript.

^abc^Statistically significant differences (P ≤ 0.05) for interaction type of fat × age of emu were marked with different letters in the upper index.

Mineral analysis of the back fat and abdominal fat composition of the 15-year-old females and males (Table 7) shows that the type of fat does not affect Pb, Cu, Ba, Cr, Cd and Sr (*P ≥ *0.05). A higher amount of other mineral components was found in back fat (*P ≤ *0.05). Gender influence was observed in the case of Si, Ca, Cu and Sr (*P ≤ *0.05), with a higher content of these elements in 15-years-old male fat. The interaction between fat type and sex of 15-years-old emu was observed for P, Ca and Fe (*P ≤ *0.05). Most P (119 mg/kg) was found in the back fat of females and Ca (18.8 mg/kg) and Fe (4.09 mg/kg) in the back fat of males.

**Table 7:** Mineral content (mg/kg) of back fat and abdominal fat of 15-years-old emu depending on sex (mean ± SE).

**Item**	**Fat type**	**Emu sex**			**Main effect**		
		**Female** **n = 8**	**Male** **n = 6**	**Average fat type**	**Type**	**Sex**	**Type × Sex**
Na	Back fat	41.7 ± 2.2	45.6 ± 2.7	43.4* ± 1.7	< 0.0001	0.8980	0.1121
	Abdominal fat	24.5 ± 1.8	21.2 ± 2.1	23.1* ± 1.4			
	Average emu sex	33.1 ± 2.6	33.4 ± 4.0	33.2 ± 2.2			
K	Back fat	134 ± 6	146 ± 7	139* ± 5	< 0.0001	0.2773	0.3654
	Abdominal fat	110 ± 4	111 ± 5	110* ± 3			
	Average emu sex	122 ± 5	128 ± 7	125 ± 4			
Ca	Back fat	15.3^b^ ± 0.5	18.8^a^ ± 0.7	16.8* ± 0.6	< 0.0001	0.0003	0.0275
	Abdominal fat	10.5^c^ ± 0.5	11.5^c^ ± 0.4	10.9* ± 0.4			
	Average emu sex	12.9_b_ ± 0.7	15.1_a_ ± 1.2	13.8 ± 0.7			
Mg	Back fat	7.15 ± 0.43	7.68 ± 0.70	7.38* ± 0.38	0.0011	0.3507	0.8452
	Abdominal fat	5.55 ± 0.35	5.89 ± 0.27	5.70* ± 0.23			
	Average emu sex	6.35 ± 0.34	6.78 ± 0.45	6.54 ± 0.27			
P	Back fat	119^a^ ± 4	105^b^ ± 5	113* ± 3	< 0.0001	0.1557	0.0140
	Abdominal fat	81.1^c^ ± 2.4	85.1^c^ ± 1.0	82.8* ± 1.5			
	Average emu sex	100 ± 5	95.1 ± 3.9	97.9 ± 3.5			
Fe	Back fat	2.97^b^ ± 0.16	4.09^a^ ± 0.30	3.45* ± 0.22	0.0002	0.2683	0.0003
	Abdominal fat	2.92^b^ ± 0.15	2.28^b^ ± 0.23	2.64* ± 0.15			
	Average emu sex	2.95 ± 0.11	3.18 ± 0.33	3.05 ± 0.15			
Cr	Back fat	0.045 ± 0.003	0.044 ± 0.003	0.044 ± 0.002	0.6916	0.6642	0.4116
	Abdominal fat	0.041 ± 0.003	0.045 ± 0.003	0.043 ± 0.002			
	Average emu sex	0.043 ± 0.002	0.044 ± 0.002	0.044 ± 0.002			
Cu	Back fat	0.076 ± 0.010	0.118 ± 0.014	0.094 ± 0.010	0.5792	0.0048	0.3904
	Abdominal fat	0.079 ± 0.008	0.103 ± 0.011	0.089 ± 0.007			
	Average emu sex	0.078_b_ ± 0.006	0.111^a^ ± 0.009	0.092 ± 0.006			
Mn	Back fat	0.0048 ± 0.0004	0.0053 ± 0.0004	0.0050* ± 0.0003	0.0222	0.2554	0.6248
	Abdominal fat	0.0041 ± 0.0002	0.0044 ± 0.0003	0.0042* ± 0.0002			
	Average emu sex	0.0044 ± 0.0002	0.0048 ± 0.0003	0.0046 ± 0.0002			
Se	Back fat	0.106 ± 0.005	0.115 ± 0.004	0.110* ± 0.003	0.0063	0.8693	0.0611
	Abdominal fat	0.101 ± 0.005	0.091 ± 0.005	0.097* ± 0.004			
	Average emu sex	0.104 ± 0.003	0.103 ± 0.005	0.104 ± 0.003			
Si	Back fat	68.0 ± 1.9	77.6 ± 2.7	72.1* ± 2.0	0.0230	0.0011	0.4639
	Abdominal fat	64.3 ± 1.9	70.7 ± 2.3	67.0* ± 1.7			
	Average emu sex	66.1_b_ ± 1.4	74.1_a_ ± 2.0	69.6 ± 1.4			
Zn	Back fat	1.09 ± 0.09	1.28 ± 0.11	1.17* ± 0.07	0.0003	0.0660	0.5547
	Abdominal fat	0.82 ± 0.04	0.92 ± 0.04	0.86* ± 0.03			
	Average emu sex	0.96 ± 0.06	1.10 ± 0.08	1.02 ± 0.05			
Ba	Back fat	0.042 ± 0.003	0.050 ± 0.005	0.045 ± 0.003	0.8328	0.1571	0.8182
	Abdominal fat	0.044 ± 0.004	0.049 ± 0.004	0.047 ± 0.003			
	Average emu sex	0.043 ± 0.003	0.050 ± 0.003	0.046 ± 0.002			
Cd	Back fat	0.022 ± 0.001	0.021 ± 0.002	0.021 ± 0.001	0.9823	0.8420	0.5759
	Abdominal fat	0.041 ± 0.003	0.045 ± 0.003	0.043 ± 0.002			
	Average emu sex	0.021 ± 0.001	0.022 ± 0.001	0.021 ± 0.001			
Pb	Back fat	0.196 ± 0.009	0.199 ± 0.010	0.197 ± 0.007	0.8970	0.7681	0.9947
	Abdominal fat	0.195 ± 0.005	0.198 ± 0.008	0.196 ± 0.004			
	Average emu sex	0.196 ± 0.005	0.198 ± 0.006	0.197 ± 0.004			
Sr	Back fat	0.016 ± 0.001	0.021 ± 0.002	0.018 ± 0.001	0.6513	0.0008	0.7068
	Abdominal fat	0.015 ± 0.001	0.021 ± 0.002	0.017 ± 0.001			
	Average emu sex	0.015_b_ ± 0.001	0.021_a_ ± 0.001	0.018 ± 0.001			

*The same symbol in the superscript indicates statistically significant differences (P ≤ 0.05) for the type of fat.

_abc_Statistically significant differences (P ≤ 0.05) for the emu age were marked with different letters in the subscript.

^abc^Statistically significant differences (P ≤ 0.05) for interaction type of fat × age of emu were marked with different letters in the upper index.

## Discussion

Our research revealed that adipose tissue water and protein content increased with age, while the fat content decreased. As is well known, the higher water content in the tissue fat contributes to its greater susceptibility to the development of pathogenic microflora and an increased tendency to rancidity. Such fat has a lower melting point, therefore its technological usefulness is limited. In turn, a higher protein content may have a reducing effect on the storage stability of the product. The scientific literature lacks information on the basic chemical composition of fatty tissue of emu and other economically used ratites. For poultry, only the basic composition of goose fat is known. Bełkot and Pyz-Łukasik^28^ studied the basic chemical composition of back fat and abdominal fatty tissue in Biała Kołudzka goose aged 16–18 weeks and 3 years. The authors of those studies stated that age significantly differentiated the amount of basic components in back fat. Higher water (15.28%), protein (2.60%) and ash (0.14%) content was found in younger geese while fat (91.5%) in older geese, i.e. contrary to our own studies (Table 2). In the studies of the quoted authors, back fat and abdominal fat differed significantly in respect of the content of all chemical components in 16–18 week old geese, whereas in 3-year-old geese only for the level of protein. Less fat (82.6%) and more water (15.3%), protein (young geese − 2.60%, older geese − 1.20%) and ash (0.14%) were found in back fat. A similar dependence on protein and fat content in back fat was found in our own studies (Tables 1 and 2). It should also be noted that in comparison to the study by Bełkot and Pyz-Łukasik^28^, the back fat of emu was characterized by a lower content of water, protein and ash, and a higher content of fat. Abdominal fat, on the other hand, contains less water, more fat and similar level of protein and ash.

In the study of Okruszek^35^ on 17-week-old Rypin geese and swan geese no influence of the breed on the fat content in abdominal fat was found, its content (97.1–97.4%) was similar to our own results. However, in subsequent studies by the same author^36^ on older 24-week-old females of the same breeds, higher fat levels were recorded (98.2–98.7%), which may suggest the opposite effect of age on fat content than in our own studies.

Fukushima et al.^37^ analysed the content of cholesterol in emu oil and recorded a value similar to that obtained in our own study for 1-year and 3-year-old male birds (Table 4). It should be added, however, that the authors did not provide the age and sex of the examined birds.

In case of 14-month-old ostriches^38^ differences in cholesterol content were found depending on the type of fat. Back fat contained more cholesterol (74.3 mg/100 g) than breast fat (49.5 mg/100 g). Those results differ from ones obtained in our own studies (Tables 3 and 4), which is probably due to species differences.

Haraf et al.^29^ noted the influence of the breed on the content of cholesterol in the abdominal fat of 17-week-old geese. The content of this sterol was 70.2 mg/100 g for Kartusian geese and 79.9 mg/100 g for Lublin geese. Comparing those results to our own studies we can conclude that emu fat has a lower cholesterol content (Tables 4 and 5).

Wang et al.^39^ did not report any significant differences in the fatty acid profile in abdominal fat and back fat of emu. Then, studies carried out by Shimizu and Nakano^40^ on back fat, peritoneal cavity fat and kidney leaf fat of emu were not supported by statistical analysis. In those studies SFA (29.8–31.5%) and MUFA (56.0–58.8%) were diametrically different from the results of our own studies (Table 4) on 1-year and 3-year-old emu males (SFA: 54.0–54.6%; MUFA: 32.4–34.4%). This was related to the double content of C16:0 and the double content of C18:1n9. The 15-year-old females and males (Table 5) had a higher SFA share of about 13% and a lower MUFA share of about 17%. It should be noted that Wang et al.^39^ and Shimizu and Nakano^40^ did not specify the birds age, sex, feeding habits and feed composition, which have a significant effect on the fatty acid profile. The content of PUFA (11.3–13.5%) and particular fatty acids (C18:2n6: 10.3–11.4%; C18:3n3: 0.90–1.86%) in the studies of the above mentioned authors was similar to the results obtained in our own studies (Tables 4 and 5).

Similar studies were conducted by Shimizu and Nakano^40^, Horbańczuk et al.^38^, Hoffman et al.^41^ and Majewska et al.^30^ on ostrich fat. The share of particular fatty acid groups in the studies of the quoted authors varied for SFA: 31.8–49.12%, for MUFA: 29.6–44.3% and for PUFA: 15.8–38.6%. The quoted authors found significant differences depending on the type of fat (back and breast fat—Horbańczuk et al.^38^; breast and abdominal fat—Hoffman et al*.*^41^; back, abdominal and breast fat—Majewska et al.^30^). Majewska et al.^30^ recorded a significantly lower share of SFA and a higher share of unsaturated fatty acids in abdominal fat. Hoffman et al.^41^ studying fat from different ostrich genetic groups (South African Black ostriches, Zimbabwean Blue Necks and their hybrids) noted many statistically significant differences in the profile of fatty acids.

In back fat of rheas, 36.9% SFA, 40.2% MUFA and 22.9% PUFA were found^40^. Comparing those results to the results obtained in our own studies, a conclusion can be drawn that the fatty acid profile of rheas is more similar to that of the 15-year-old emus rather than that of the 1-year and 3-year-old birds (Tables 4 and 5).

Numerous studies on goose fat have shown that the content of fatty acid groups can vary over a wide range: SFA—19.45–33.41%; MUFA—41.90–67.32%; PUFA—11.27–32.95%^28,29,31,36,42–45^. These differences are due to the fact that the research was carried out on geese of various breeds and ages. This also explains the apparent differences in SFA and MUFA content compared to our own research, in particular to 1-year and 3-year emus (Tables 4 and 5). Additionally, it should be mentioned that the changes in the fatty acid profile of goose fat are influenced by nutrition^31,42,43^, breed^29,35,36^, packing time and conditions^44^, age^28,42^ and type of fat^28^.

Although the content of elements in animal raw materials is very important, the scientific literature lacks information on their content in the spare tissue of animals. In general, too high mineral content in fats is undesirable because of the reduction of their durability due to the catalytic effect of metals on the process of fat oxidation during storage and processing. The quality of fat and oil in terms of their storage and toxicity can be assessed by determining the content of certain elements. This applies especially to Fe, Cu, Mg, Co, Zn, Mn and Ni^46,47^. According to our research, back fat contains more of the metals responsible for the catalytic processes of fat oxidation. The content of most of these elements increased with age of the emus. On the other hand, elements such as As, Cr, Cd and Pb are undesirable due to their toxicity and adverse role in the body metabolism^46,47^. Our own research shows that the accumulation of these elements is not affected by the type of body fat. However, the effect of age is noticeable only during longer production cycle (15 years).

Codex Standard for Named Animal Fats CODEX-STAN 211–1999 (FAO/WHO) gives the permitted limit for certain elements in edible animal oils. It shows that the tested emu fat (Tables 6 and 7) did not exceed the permissible limit for Cu (max 0.4 mg/kg) and As (max 0.1 mg/kg), which was below the detection level. Fat from 15-year-old emus exceeded the maximum limit for Pb (max 0.1 mg/kg) twice, which is most likely related to the age of the bird. The content of Pb in the 1-year and 3-year-old emus was within the permissible standard. In case of Fe (max 1.5 mg/kg) only the 3-year-old emus did not exceed the maximum permissible level of this element. It should be noted, however, that emu fat is only the raw material from which the oil is produced and it can be assumed that only part of the minerals will enter the oil, which is related to the method of its extraction. Ponphaiboon et al.^48^ proved that the content of iron and polyunsaturated fatty acids in ostrich oil and its antioxidant activity were dependent on the method of oil extraction. Therefore, we propose that an appropriate extraction method could also eliminate the excess of undesirable elements from the fat of birds kept longer, so that they meet the acceptable standards and could also be used.

## Conclusions

The conducted research is a source of knowledge on the chemical composition of emu fat, which is a valuable raw material in the pharmaceutical, cosmetic and food industries. Slight differences in the basic chemical composition, fatty acid profile depending on the location of adipose tissue in the bird's body indicate that emu both dorsal and abdominal fats are a valuable raw material for processing and there is no need for their separation in the technological process.

Fat of 15-year-old emu, regardless of the type, was characterized by the largest share of unsaturated fatty acids (MUFAs and PUFAs). From a nutritional point of view, this is a desirable trait, though it can be problematic for the technological process, especially in terms of accelerating rancidification. In addition, 15-year-old emu fat had a higher content of heavy metals. To sum up, fat obtained from young, 1- and 3-year-old birds seems to be most recommendable for use in the food and cosmetics industries. Research on the quality of this raw material should continue, with attention to oxidative stability and sensory quality.

## Data Availability

All data generated or analyzed during this study are included in this published article.
